# Combined [^18^F]DPA-714 micro-positron emission tomography and autoradiography imaging of microglia activation after closed head injury in mice

**DOI:** 10.1186/s12974-016-0604-9

**Published:** 2016-06-07

**Authors:** Ina Israel, Andrea Ohsiek, Ehab Al-Momani, Christiane Albert-Weissenberger, Christian Stetter, Stine Mencl, Andreas K. Buck, Christoph Kleinschnitz, Samuel Samnick, Anna-Leena Sirén

**Affiliations:** Department of Nuclear Medicine, University Hospital Würzburg, 97080 Würzburg, Germany; Experimental Neurosurgery, Department of Neurosurgery, University Hospital Würzburg, 97080 Würzburg, Germany; Department of Neurology, University Hospital Würzburg, 97080 Würzburg, Germany; Department of Neurology, University Hospital Essen, 45147 Essen, Germany

**Keywords:** Neuroinflammation, TBI, IBA-1, Immunohistochemistry, Focal, Diffuse, Weight drop, TSPO, PET, Autoradiography

## Abstract

**Background:**

Traumatic brain injury (TBI) is a major cause of death and disability. Neuroinflammation contributes to acute damage after TBI and modulates long-term evolution of degenerative and regenerative responses to injury. The aim of the present study was to evaluate the relationship of microglia activation to trauma severity, brain energy metabolism, and cellular reactions to injury in a mouse closed head injury model using combined in vivo PET imaging, ex vivo autoradiography, and immunohistochemistry.

**Methods:**

A weight-drop closed head injury model was used to produce a mixed diffuse and focal TBI or a purely diffuse mild TBI (mTBI) in C57BL6 mice. Lesion severity was determined by evaluating histological damage and functional outcome using a standardized neuroscore (NSS), gliosis, and axonal injury by immunohistochemistry. Repeated intra-individual in vivo μPET imaging with the specific 18-kDa translocator protein (TSPO) radioligand [^18^F]DPA-714 was performed on day 1, 7, and 16 and [^18^F]FDG-μPET imaging for energy metabolism on days 2–5 after trauma using freshly synthesized radiotracers. Immediately after [^18^F]DPA-714-μPET imaging on days 7 and 16, cellular identity of the [^18^F]DPA-714 uptake was confirmed by exposing freshly cut cryosections to film autoradiography and successive immunostaining with antibodies against the microglia/macrophage marker IBA-1.

**Results:**

Functional outcome correlated with focal brain lesions, gliosis, and axonal injury. [^18^F]DPA-714-μPET showed increased radiotracer uptake in focal brain lesions on days 7 and 16 after TBI and correlated with reduced cerebral [^18^F]FDG uptake on days 2–5, with functional outcome and number of IBA-1 positive cells on day 7. In autoradiography, [^18^F]DPA-714 uptake co-localized with areas of IBA1-positive staining and correlated strongly with both NSS and the number of IBA1-positive cells, gliosis, and axonal injury. After mTBI, numbers of IBA-1 positive cells with microglial morphology increased in both brain hemispheres; however, uptake of [^18^F]DPA-714 was not increased in autoradiography or in μPET imaging.

**Conclusions:**

[^18^F]DPA-714 uptake in μPET/autoradiography correlates with trauma severity, brain metabolic deficits, and microglia activation after closed head TBI.

## Background

Traumatic brain injury (TBI) due to falls or accidents in traffic and during sport activities is the leading cause of disability and death among young adults and children in Europe and the USA [1, 2]. Furthermore, traumatic insults to the brain due to falls have become an increasing health problem in the elderly [2, 3]. Even though improved emergency and hospital care have reduced the acute mortality of TBI, surviving patients often live with severe disabilities and develop progressive brain damage or dementia of unknown origin [3]. Neuroinflammation is a key event that contributes to chronic neurodegeneration and demyelination, prevents regeneration, and causes functional deficits after TBI [4, 5]. As the inflammatory responses after TBI are complex and probably reflect the heterogeneity of injury mechanism and comorbidities in the TBI population [3–5], a better characterization of the spatial and temporal evolution of inflammation by repeated intra-individual monitoring is needed for the development of targeted therapies. In this context, repeated in vivo positron emission tomography (PET) imaging of neuroinflammation represents a powerful tool and in combination with autoradiography enables resolution at the cellular level [5–11].

Microglia, the brain macrophages, are the first line of defense against brain injuries. In TBI, resident microglia are rapidly mobilized to the injury site, where they contribute to acute damage and modulate the long-term evolution of degenerative and regenerative responses to injury [4, 8, 12, 13]. Therefore, microglial cell-surface and mitochondrial receptors have been used as specific targets in the development of diagnostic biomarkers of neuroinflammation. One of these microglial targets is the 18-kDa translocator protein TSPO, a protein of the outer mitochondrial membrane that is specifically upregulated in activated microglia in injured brain and in neurodegenerative disease associated with neuroinflammation [8, 14].

The radiotracer [^11^C]PK11195 has been most commonly used as radiolabeled TSPO ligand for in vivo PET imaging or autoradiography [6, 14–16]. However, the major drawback of PET with [^11^C]PK11195 is the high unspecific accumulation of [^11^C]PK11195 in healthy brain resulting in a poor signal to noise ratio [16]. Therefore, efforts have been undertaken to develop a more specific radiotracer than [^11^C]PK11195 for molecular imaging of TSPO by PET. Among the recently developed radiotracers for targeting TSPO, the radiofluorinated agent N,N-diethyl-2-(2-(4-(2-[^18^F]fluoroethoxy)phenyl)-5,7-dimethyl-pyrazolo[1,5-α]pyrimidin-3-yl)acetamide ([^18^F]DPA-714) exhibited superior binding affinities, low lipophilicity, and an improved target-to-background binding ratio in previous comparative investigations [16, 17]. Moreover, the longer physical half life (t½) of ^18^F of 110 min, compared with t½ = 20 min for ^11^C makes [^18^F]DPA-714 highly suitable for PET imaging even in facilities without an in-house cyclotron. Due to these advantages, [^18^F]DPA-714 has been tested experimentally as an imaging probe for TSPO in different models [11, 16–18]. In the present study, we aimed at evaluating microglia activation in a murine closed head injury model using a combined approach of in vivo [^18^F]DPA-714 PET imaging, combined ex vivo autoradiography, and IBA-1 immunohistochemistry. In particular, we wanted to determine the relationship of microglia activation to trauma severity and cellular reactions to injury and correlate TSPO ligand binding to brain glucose uptake in the first 2–5 days after trauma by 2-[^18^F]Fluoro-2-deoxyglucose ([^18^F]FDG) PET imaging.

## Methods

### Radiochemistry

All chemicals and solvents were purchased commercially from Sigma-Aldrich (Deisenhofen, Germany) and Carl Roth (Karlsruhe, Germany), respectively. If not stated otherwise, they were used in the synthesis without further purification.

[^18^F]Fluoride for the synthesis of N,N-diethyl-2-(2-(4-(2-[^18^F]fluoroethoxy)phenyl)-5,7-dimethyl-pyrazolo[1,5-α]pyrimidin-3-yl)acetamide ([^18^F]DPA-714) and [^18^F]FDG was produced on the GE-PETtrace cyclotron (General Electric Medical Systems, Uppsala, Sweden) at the Interdisciplinary PET-Centre (IPZ) of the University Hospital of Würzburg via ^18^O(p,n)^18^F reaction by irradiating 3.0 mL of 97 % enriched [^18^O]H_2_O with 16.5 MeV protons. [^18^F]FDG and [^18^F]DPA-714 were synthesized using a GE Fastlab® synthesis module (GE Medical Systems, Uppsala, Sweden) and the Raytest SynChrom module (Raytest, Straubenhardt, Germany), respectively as described previously [19]. Both radiotracers have been established for clinical and preclinical applications.

### Animal model

All animal experiments were carried out according to the Guide for the Care and Use of Laboratory Animals published by the US National Institutes of Health (NIH Publication No. 85-23, revised 1996) and in compliance with the German animal protection law. Experiments were approved by the district government of Lower Franconia (Regierung von Unterfranken AZ: 55.2/2531.01-53/12). Male 10–15-week-old C57BL/6N mice were purchased from Charles River (Sulzfeld, Germany) and maintained in the animal facility of the University of Würzburg, Department of Neurology. A closed head traumatic brain injury was produced as previously described [20–22] in 43 mice. Briefly, the mice were anesthetized and maintained with 2 % isoflurane anesthesia in 100 % oxygen during the whole procedure. A midline longitudinal scalp incision was made to expose the skull. The head was fixed by holding it with two fingers to keep the mice in the right position and to allow a slight movement at the moment of the trauma induction. After identification of the impact area over the right fronto-parietal cortex, TBI was induced by a falling weight (95 g) with a silicone-covered blunt tip onto the skull from the height of 3 cm (*n* = 14, 8 classified as mTBI, 6 classified as TBI) to 4 cm (*n* = 29, 16 classified as mTBI and 19 classified as TBI). After TBI induction, the mice shortly received 100 % oxygen. The skull was examined to preclude fractures and the skin closed. Sham operation included anesthesia and exposure of the skull but without weight-drop injury. It was performed in 10 mice. The neurobehavioral status of the mice was assessed by the neurological severity score (NSS) [21, 23, 24], a composite score including tasks on motor function, alertness, and physiological behavior, with higher scores indicating more severe deficit. Functional testing was performed initially at 1 h after and repeated on days 1 and 7 after weight-drop injury by investigators blinded to the experimental groups.

Final TBI classification was based on postmortem evaluation of morphological damage on hematoxylin-eosin stained sections. The mice were sacrificed by CO_2_ inhalation, and the brains were quickly removed and immediately frozen in methyl butane on dry ice. Coronal 18 μm-thin sections throughout the complete forebrain were cut in a freezing microtome (Leica CM3050 S cryostat, Leica, Wetzlar, Germany) and mounted on microscope slides. The brain sections were fixed with 4 % paraformaldehyde and stained with hematoxylin-eosin using standard protocols. Damage including focal cortical contusions was used as criteria for trauma severity; all cases without focal lesions were classified as mild injuries (mTBI), cases with focal lesions as TBI.

### μPET studies

The [^18^F]DPA-714-μPET scans were performed and evaluated in 15 mice on day 1, 28 mice on day 7, and 6 mice on day 16 after TBI. A [^18^F]FDG-μPET scan was acquired between days 2–5 after TBI in 28 mice. μPET scans and image analysis were carried out using the Inveon μPET scanner (Inveon®, Siemens Medical Solutions, Knoxville, TN, USA). Mice were kept under 1.5 % isoflurane anesthesia in 100 % oxygen during the whole time between tracer injection and the end of the PET scan. Their body temperature was maintained at physiological level with a custom-made heating pad during the procedure. The animals received an injection of 7.4 ± 4.6 MBq of [^18^F]DPA-714 or 5.8 ± 1.5 MBq of [^18^F]FDG in a volume of 100-150 μL into the tail vein. In case of [^18^F]FDG-μPET food was removed 4 h before injection, water was available anytime. The mice were then placed in prone position into the animal scanner bed to perform a 20 min μPET scan 40 min after [^18^F]DPA-714 administration and 10 min μPET scan 50 min after administration of [^18^F]FDG, respectively.

The acquired 3D dataset was sorted with Fourier rebinning (FORE) to a 2D dataset of sinograms, which were reconstructed with the OSEM2D reconstruction algorithm. We used the software AMIDE Medical Image Data Examiner (Version 1.0.4) to quantify the radioactivity uptake into different regions of interest (ROI). For the semi-quantitative analysis, PET images of each animal were manually co-registered and two spherical ROI were defined and used as a template for all images of the same animal. The first ROI was outlined into the region with the highest [^18^F]DPA-714 uptake on day 7 after TBI. In the case that no increase in [^18^F]DPA-714 uptake was detectable on day 7 (e.g., in the sham group), the ROIs were drawn in the same brain region as those of a co-registered TBI mouse showing increased [^18^F]DPA-714 uptake in the damaged brain tissue.

The second ROI was placed into the cerebellum, which was used as a relative reference tissue. We choose cerebellum as a relative reference area, since in our TBI model diffuse cellular damage after weight drop involves forebrain structures in both the ipsilateral and contralateral cerebral hemispheres whereas hindbrain and cerebellum appear normal in standard histology. Moreover, the cerebellum has been recently validated as a pseudo-reference region for TSPO binding radiotracers in Alzheimer patients [25] and in a mouse model of Alzheimer’s disease for both TSPO ligands and [^18^F]FDG PET [26]. It has also been used as internal reference for assessment of brain metabolism with [^18^F]FDG PET in rat models of focal TBI [27, 28]. To ensure, that cerebellum can be defined as a reference region for our TBI model, we calculated the mean standardized uptake value (SUV) for the outlined ROI of cerebellum in all examined mice and performed a statistical evaluation according to the differences in the cerebellar [^18^F]DPA-714 uptake between sham, mTBI, and TBI groups during all time-points after trauma induction. As no statistically significant differences were found between the three groups, at any time point (*p* = 0.62), we concluded that cerebellum can be considered as a stable reference region also in our model. Finally, the [^18^F]DPA-714 uptake values of the region with the highest tracer uptake were divided by the uptake values of the ROI of the cerebellum and referred to as lesion-to-cerebellum ratio (L/C ratio).

### Ex vivo autoradiography and concomintant IBA-1 immunohistochemistry

Combined autoradiography and immunostaining for IBA-1 was performed subsequent to the [^18^F]DPA-714-μPET scan on day 7 in 5 sham-operated, 11 mTBI, and 6 TBI mice and on day 16 in 3 mTBI and 3 TBI mice. After the μPET-scans (60 min after injection of [^18^F]DPA-714), the mice were sacrificed by CO_2_ inhalation. Brains were harvested and immediately frozen in methyl butane on dry ice. Coronal sections (18 μm) were cut on microscope slides in a freezing microtome (Leica) as described above. The sections were exposed on a phosphor image plate (Biostep, Jahnsdorf, Germany) overnight. The image plate was read out on a image plate scanner (Dürr Medical, Bietigheim-Bissingen, Germany). After imaging, the same sections were processed for IBA-1 immunohistochemistry as described below.

For quantification, one circular ROI was placed in the area of highest [^18^F]DPA-714 accumulation representing the highest microglia activation and a second circular ROI was drawn in the area of lowest [^18^F]DPA-714 uptake and the lowest microglia activation. As the weight-drop trauma generates a variable pattern of focal lesions and diffuse axonal damage in both cortical hemispheres, the regions of interest could not always be set in the same position. To ensure the correct ROI position in the region of the highest and lowest microglia activation, each autoradiography image was compared with the staining pattern of IBA-1-labelled microglia on the same cryosection. For semi-quantitative data analysis, [^18^F]DPA-714 uptake values of the region with the highest microglia activation were divided by the uptake values of the region with the lowest microglia activation and referred to as TBI-to-normal-tissue-ratio (TBI/N ratio). Data analysis and image editing were performed with the software AMIDE Medical Image Data Examiner (version 1.0.4) and Gimp (cersion 2.8.14), respectively.

### In vitro autoradiography

In a separate study, mice (*n* = 15) were euthanized 7 days after TBI and the brains removed for cryosectioning for correlative assessment of [^18^F]DPA-714 uptake to histological damage and cellular reactions on adjacent brain sections. Eighteen-micrometer cryosections were prepared as described above and washed in PBS, dryed, and subsequently incubated with 100 μL [^18^F]DPA-714 (13 MBq/mL) for 30 min at room temperature (RT). Afterwards, the cryosections were washed three times in PBS and finally shortly rinsed in deionized water. After drying, the sections were exposed on a phosphor image plate (Biostep, Jahnsdorf, Germany) for 30 min. The image plate was read out on a image plate scanner (Dürr Medical, Bietigheim-Bissingen, Germany).

### Immunohistochemistry

The cryosections were fixed with 4 % paraformaldehyde and subsequently washed in PBS, blocked with 10 % normal horse serum (NHS) (Jackson Immuno Research, Suffolk, UK) and 0.2 % Triton X. After a further washing step with PBS, the tissue was incubated with primary antibodies in PBS with 2 % NHS and 0.5 % Triton X for 2 days at 4 °C. The sections were washed in PBS and incubated with biotinylated second antibodies in PBS with 2 % NHS and 0.5 % Triton X for 1 h at RT. After washing in PBS, sections were incubated with Avidin-Biotin (Standard Ultra Sensitive ABC Staining Kit, Thermo Scientific, Rockford, IL, USA) for 30 min at RT and subsequently incubated for 4 min with 3,3′-diaminobenzidine (DAB) or DAB with nickel enhancement for all IBA-1 stained sections. The reaction was stopped in water, and the tissue was dehydrated using an increasing ethanol series and xylol. Finally, the sections were mounted with coverslips using Vitro Clud (R. Langenbrinck, Emmendingen, Germany) embedding medium. The following antibodies were used: polyclonal rabbit anti-IBA-1 (1:5000, WAKO Pure Chemical Industries, Neuss, Germany), monoclonal mouse anti-glial fibrillary acidic protein (GFAP, 1:1000, Novocastra, Newcastle upon Tyne, UK), and monoclonal mouse anti-non-phosphorylated neurofilament H (SMI-32, 1:1000, Covance, Freiburg, Germany), biotinylated goat anti-rabbit or horse anti-mouse IgGs (Vector Laboratories, Burlingame, CA, USA).

### Quantification of immunohistochemical staining

IBA-1 staining on the cryosections used first for ex vivo [^18^F]DPA-714 autoradiography was assessed under a light microscope (Olympus BH2, Olympus-Germany GmbH, Hamburg, Germany) using an objective with ×40 magnification. Successful staining that could be quantified was obtained in 22 mice on day 7 and in 5 mice on day 16. The numbers of IBA-1 positive microglia were determined from four cortical fields in the ipsilateral and contralateral hemispheres in four separate brain sections per animal and expressed as mean cell count/mm^2^ for each animal.

In a separate set of 15 mice used for in vitro autoradiography, the number of GFAP-positive astrocytes and SMI-32-positive retraction bulbs as a marker for damaged axons [29–31] were counted in adjacent sections to those used for [^18^F]DPA-714 autoradiography on day 7. GFAP-positive astrocytes were counted on four cortical fields in three separate brain sections per animal and expressed as mean cell count/mm^2^. SMI-32-positive retraction bulbs were counted in both brain hemispheres in three separate brain sections per animal and expressed as mean counts/section for each animal.

### Statistical analysis

The NSS scales are depicted as scatter plots, including median with the 25 percentile and the 75 percentile given in parentheses in the text. All other data are expressed as mean ± standard deviation (SD). The statistics were performed by using PrismGraph 5.0 software package (GraphPad Software, GraphPad Inc, La Jolla, CA, USA). Data were tested for Gaussian distribution and homogeneity of variance with the Kolmogorov-Smirnov test and the Levene’s test, respectively. In case of normal distribution and variance homogeneity (PET), the statistical significance was tested by one-way analysis of variance (ANOVA) with post hoc Bonferroni test.

Non-parametric data (NSS, autoradiography) were analyzed using Kruskal-Wallis test with post hoc Dunn’s correction. For linear regression analysis the Spearman’s Rho rank correlation (*r*_s_) was calculated. *p* values < 0.05 were considered statistically significant.

## Results

### Radiochemistry

[^18^F]DPA-714 and [^18^F]FDG were prepared in-house at the Interdisciplinary PET-center of the University Würzburg. [^18^F]DPA-714 was obtained in 65–73 % radiochemical yield and a specific activity of 78 ± 35 GBq/μmol using a Raytest Synchrom module. [^18^F]FDG was obtained in 85–87 % radiochemical yield following the GE Fastlab methodology, as routinely prepared for clinical applications. Before use, [^18^F]DPA-714 and [^18^F]FDG were analyzed by HPLC for radiochemical purity and sterile filtered through a 0.22-μm sterile filter. Radiochemical purity was ≥99 % for both radiopharmaceuticals.

### Functional outcome and lesion severity

**Fig. 1:**
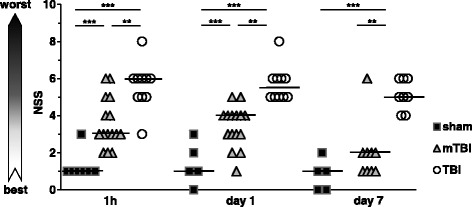
Functional outcome of mice after closed head weight-drop TBI. Outcome was assessed by using the neurological severity score (NSS), a composite score including tasks on motor function, alertness, and physiological behavior, with higher scores indicating worse deficit. *Black squares* indicate sham-operated mice (*n* = 7 on 1 h and on day 1, *n* = 5 on day 7), *gray triangles* mTBI (*n* = 15 1 h and on day 1, *n* = 9 on day 7), and *open circles* TBI mice (*n* = 10 1 h and on day 1, *n* = 8 on day 7), ***p < 0.001, **p < 0.01)

Functional deficits on the neuroscore (NSS) in all TBI mice were significantly worse than in sham-operated mice over the first 24 h (Fig. 1). The initial NSS (median, 25 %, 75 % percentile) 1 h after trauma was 6 (5, 6, *n* = 10) in mice with focal lesions in postmortem histology; in mTBI mice without such focal defects, it was 3 (3, 5, *n* = 15) and in sham-operated mice 1 (1, 1, *n* = 7). On day 1 after injury, the corresponding scores were 5.5 (5, 6, *n* = 10) in TBI, 3 (3, 4, *n* = 15) in mTBI, and 1 (1, 2, *n* = 7) in sham-operated mice. On day 7, NSS in TBI mice (5, 4,25, 6, *n* = 8) was still significantly worse than in mTBI mice (2, 1, 2, *n* = 9) or in sham-operated mice (1, 0, 1.5, *n* = 5) (Fig. 1). The presence of focal cortical damage in postmortem histology correlated positively with the severity of the functional deficit on NSS 1 h after trauma (r_s_ = 0.61, *p* = 0.001, *n* = 25), on day 1 (*r*_s_ = 0.81, *p* < 0.0001, *n* = 25), and on day 7 (r_s_ = 0.73, *p* < 0.001, *n* = 17).

### μPET-studies

**Fig. 2:**
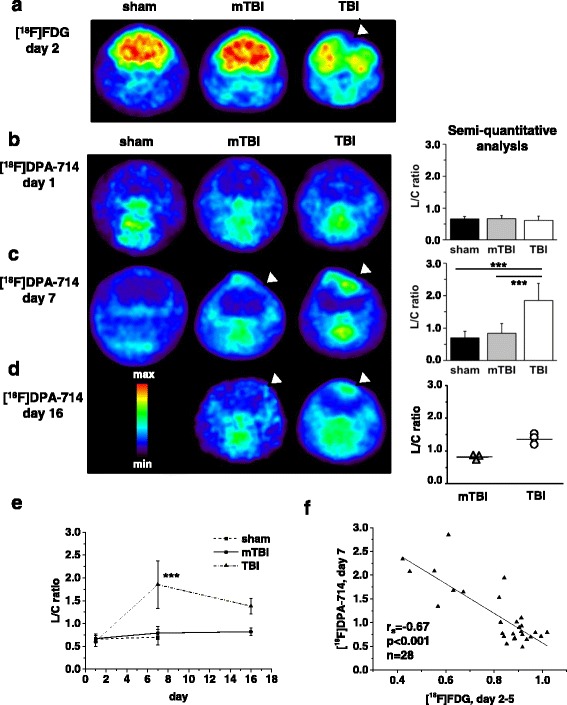
[^18^F]FDG- and [^18^F]DPA-714-μPET imaging after closed head weight-drop TBI in mice. **a** Representative transverse images of [^18^F]FDG-μPET, 2 days after TBI show an area of no [^18^F]FDG uptake within the focal cortical lesion of a TBI mouse (*white arrow head*), in contrast to a uniform [^18^F]FDG uptake in sham-operated and mTBI mice. **b**–**d** Representative transverse images of ^18^F]DPA-714-μPET on the *left* and semi-quantitative analyses of these data on the *right. Arrowheads* in the μPET images in panels. **c**, **d** The visible overlap of [^18^F]DPA-714 uptake on days 7 and 16 with the reduction of the [^18^F]FDG uptake on day 2 (**a**). **b** [^18^F]DPA-714-μPET on day 1 after TBI shows no significant difference in [^18^F]DPA-714 uptake between sham (*n* = 5), mTBI (*n* = 12), and TBI (*n* = 8) mice. **c** [^18^F]DPA-714-μPET on day 7 after TBI. Compared to the sham animals (0.7 ± 0.2, mean ± SD, *n* = 6), the lesion-to-cerebellum (L/C) ratio was not significantly increased in mTBI mice (0.8 ± 0.3, *n* = 12), but it was significantly increased in TBI mice (1.9 ± 0.5, *n* = 10) as compared to both sham and mTBI mice. **d** [^18^F]DPA-714-μPET on day 16 after TBI. The L/C ratio of three mTBI and three TBI mice. **e** Time-course of the L/C ratio in the three groups (data identical to bar graphs in **b**–**d**. The L/C ratio of TBI mice significantly increased from 0.61 on day 1 to 1.85 on day 7 as compared to both sham-operated and mTBI mice and the decreased to 1.38 on day 16. In mTBI mice the L/C ratio was not statistically different from that in sham group even if it tended to increase from 0.67 on day 1 to 0.82 on day 16. **f** Linear regression analysis between [^18^F]FDG uptake on days 2–5 and [^18^F]DPA-714 accumulation on day 7 show a highly significant correlation between reduced brain metabolic activity and [^18^F]DPA-714 uptake.***p < 0.001 in **c** and **e**

TSPO ligand binding was determined by [^18^F]DPA-714 μPET on days 1, 7, and 16 and correlated to metabolic imaging with [^18^F]FDG-μPET on day 2, 4, or 5 after TBI. As shown in the representative images in Fig. 2a, brain glucose uptake was globally reduced with a noticeable focal deficit of [^18^F]FDG accumulation in the cortical contusions on day 2 after TBI. Such deficits were not visible in mTBI or sham-operated mice (Fig. 2a). Brain uptake of [^18^F]DPA-714 in all mice was low on day 1 after TBI as illustrated in the representative images (Fig. 2b). Accordingly, the semi-quantitative analysis of the lesion-to-cerebellum (L/C) ratios on day 1 after TBI revealed no differences in radiotracer uptake between the sham-operated, mTBI, and TBI mice (Fig. 2b). On days 7 and 16, after trauma [^18^F]DPA-714 uptake was substantially increased only in cortical areas of low regional glucose uptake in μPET on day 2 (see arrowheads in the μPET images of Fig. 2). Brain [^18^F]DPA-714 uptake in mTBI or sham-operated mice remained low on das 7 (Fig. 2c). The semi-quantitative L/C ratios in TBI mice with focal lesions were 2.6-fold (*p* < 0.001) higher than in sham mice and 2.3-fold (*p* < 0.001) higher than in mice with mild TBI on day 7 (Fig. 2e). Linear correlation analysis between glucose metabolism and TSPO ligand binding revealed a highly significant correlation between [^18^F]FDG uptake on days 2, 4, and 5 and the [^18^F]DPA-714 uptake on day 7 in individual mice (Fig. 2f). On day 16, after trauma [^18^F]DPA-714 uptake into the focal contusions in TBI mice remained elevated (Fig. 2d). As only three animals in each group were available for PET-analysis on day 16, these data are shown as scatter plots of individual values with means in Fig. 2d and no statistical analysis of these data is shown.

### Ex vivo autoradiography and immunohistochemistry studies

**Fig. 3:**
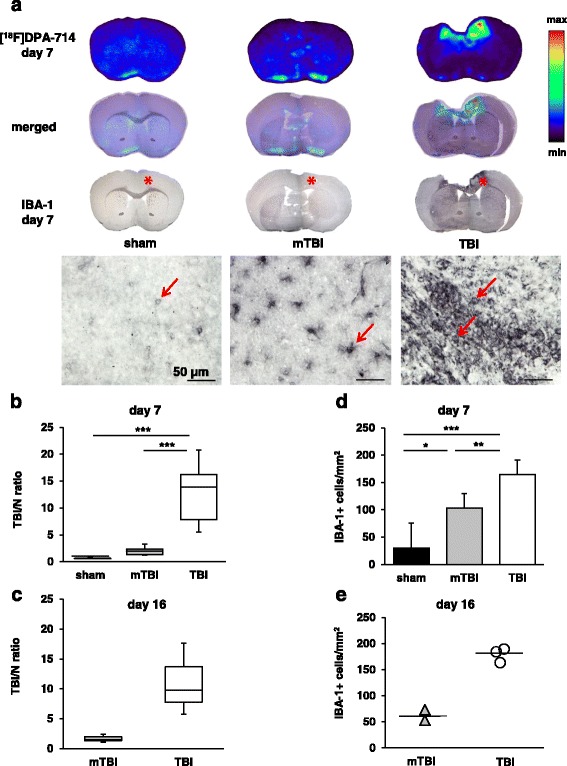
Combined ex vivo autoradiography and IBA-1 immunohistochemistry show increased [^18^F]DPA-714 uptake in microglia after closed head weight-drop TBI in mice. **a** Representative images of [^18^F]DPA-714 autoradiography and IBA-1 immunohistochemical staining on the same section of a sham, mTBI, and TBI mouse on day 7. The *middle panel* show merged images in each case. *Red asterisks* on the low magnification images denote areas depicted in higher magnification below each image. *Red arrows* mark IBA-1 positive cells, *scale bar* = 50 μm. **b** Box-plots representing calculated TBI/N ratios of [^18^F]DPA-714 binding in sham operated (*n* = 5), mTBI (*n* = 11), and TBI (*n* = 6) mice on day 7 and in **c** on day 16 in three mTBI and three TBI mice. **d** IBA-1 positive cell counts on day 7 were derived from the sections used for calculation of the TBI/N ratios in **b** in sham (*n* = 5), mTBI (*n* = 11), and TBI (*n* = 6) mice. **e** show IBA-1 counts on day 16 in two mTBI and three TBI mice used for TBI/N ratio in **c**. ***p < 0.001, **p < 0.01, *p < 0.05

Combined autoradiography and immunostaining was successful on brain sections from 5 sham-operated mice, 11 mTBI, and 6 TBI mice on day 7 and on sections of 2 mTBI mice and 3 TBI mice on day 16. In agreement with the μPET findings, autoradiography performed after μPET acquisition on day 7 and day 16 showed very low [^18^F]DPA-714 uptake into brain tissue on day 7 in sham and mTBI mice whereas in TBI mice with focal lesions intense [^18^F]DPA-714 accumulation was detectable in areas overlapping cortical lesions (Fig. 3a). In order to visualize the cellular identity of structures binding [^18^F]DPA-714, the brain sections exposed to ex vivo autoradiography were additionally stained with the microglia/monocyte marker IBA-1. As depicted in the representative images in Fig. 3a, a light IBA-1 staining was present in cells of microglial morphology in sham mice. In mTBI mice, an intensified IBA-1 staining was evident in microglia with enlarged cell body and processes. In TBI mice with focal lesions, IBA-1 staining was seen in activated microglia with mixed morphology with the most intense staining in ameboid cells directly overlapping the areas of intense [^18^F]DPA-714 binding in autoradiography (Fig. 3a).

The TBI/N ratio analysis of the autoradiography images showed in TBI mice with focal brain lesions an intense [^18^F]DPA-714 accumulation on day 7 with a significantly elevated mean TBI/N ratio of 12.8 ± 6.0 (mean ± SD, *n* = 11, *p* < 0.001) compared to sham (1.0 ± 0.02, *n* = 5) or mTBI (1.9 ± 0.7, *n* = 6) (Fig. 3b). The median (25 %, 75 % percentile) TBI/N ratio in the three TBI mice monitored until day 16 was 9.8 (7.8, 13.7) and in the mTBI mice 1.5 (1.3, 1.9). As only three animals in each group were available for analysis on day 16 (Fig. 3c), no statistical analysis of these data is shown.

Due to its higher sensitivity, avidin-biotin coupled second antibodies and DAB-enhanced immunohistochemistry rather than immunofluorescent-labelled second antibodies were used for visualization of the IBA-1 antibody staining [32]. In view of the high variability in background staining inherent to this method, automated image analysis of areas of IBA-1 immunopositive staining was not used to obtain quantitative estimates of the cellular responses. Instead, we counted the number of IBA-1 positive cells in four anatomically comparable fields of both brain hemispheres in four individual brain sections in each animal. As shown in Fig. 3d, counts of IBA-1 positive cells on day 7 were higher than in sham-operated mice in both mTBI (103 ± 27 cells/mm^2^, *p* < 0.05, *n* = 11) and TBI (165 ± 26 cells/mm^2^, *p* < 0.001, *n* = 6) mice.

Highest cell counts were found in brain sections of TBI mice with a strong [^18^F]DPA-714 binding in autoradiography on both days 7 and 16 (Fig. 3b–e).

### Correlation of [^18^F]DPA-714 brain uptake with outcome and IBA-positive cell counts

**Fig. 4:**
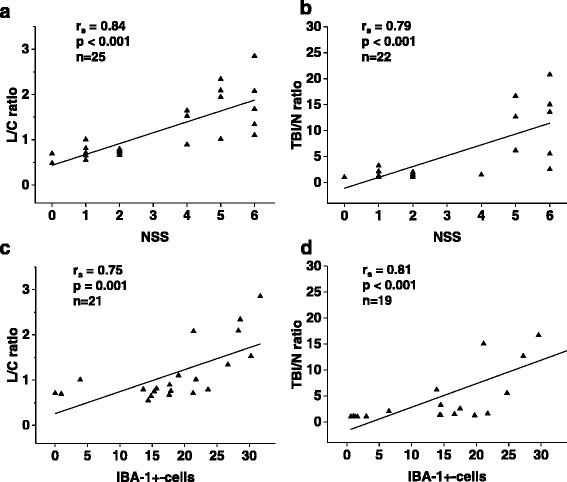
Correlation of [^18^F]DPA-714 brain uptake with outcome and IBA-positive cell counts after closed head weight-drop TBI in mice. Linear regression analysis between NSS and L/C ratios of [^18^F]DPA-714 accumulation in μPET (**a**), between NSS and TBI/N ratios of [^18^F]DPA-714 accumulation in ex vivo autoradiography (**b**), between IBA-1 counts and L/C ratios of [^18^F]DPA-714 (**c**), and between IBA-1 counts and TBI/N ratios of [^18^F]DPA-714 (**d**)

We used linear regression analysis to evaluate the correlation of [^18^F]DPA-714 brain uptake with functional outcome (NSS) and IBA-1 staining. As shown in Fig. 4) NSS on day 7 correlated strongly with both the calculated L/C ratio in μPET (Fig. 4a) and the TBI/N ratio in autoradiography (Fig. 4b). Also, the cell counts of IBA-positive cells on day 7 showed a strong correlation with both the L/C ratio in μPET (Fig. 4c) and with the TBI/N ratio in ex vivo autoradiography (Fig. 4d).

### Astrogliosis and diffuse axonal injury in relation to [^18^F]DPA-714 binding

**Fig. 5:**
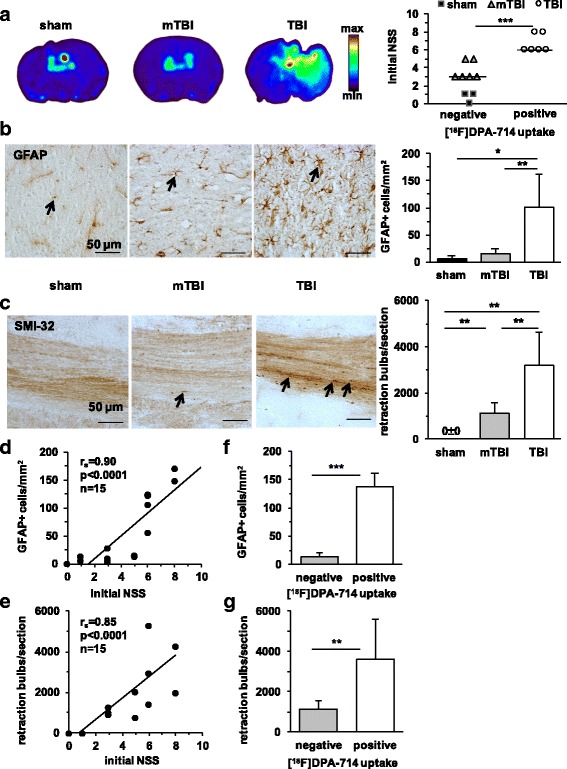
Astrogliosis and diffuse axonal injury in relation to in vitro binding of [^18^F]DPA-714 on day 7 after closed head weight-drop TBI in mice. **a** Representative images of [^18^F]DPA-714 in vitro autoradiography and the initial trauma severity (NSS at 1 h after TBI) plotted in relation to positive/negative [^18^F]DPA-714 accumulation. **b**, **c** Representative images of GFAP-positive astrocytes (**b**) or SMI-32 staining of axons (**c**) on the left and on the right counts of GFAP-positive cells or SMI-32 positive damaged axons (retraction bulbs/brain section) in sham-operated (*n* = 4), mTBI (*n* = 6), and TBI (*n* = 6) mice. *Scale bar* = 50 μm. **d**, **e** Linear regression analyses between the initial trauma severity and number of GFAP-positive cells (**d**) or SMI-32 positive retraction bulbs (**e**). **f**, **g** GFAP-positive cells counts (**f**) or damaged axons (**g**) plotted in relation to [^18^F]DPA-714 uptake in TBI mice by in vitro autoradiography (negative *n* = 6, positive *n* = 6). ***p < 0.001, **p < 0.01, *p < 0.05

Additional immunohistochemical studies were performed to evaluate astrocytic responses and axonal damage in relation to functional outcome and [^18^F]DPA-714 binding by in vitro autoradiography on day 7 after TBI in 3 sham-operated mice, 6 mTBI, and 6 TBI mice (Fig. 5). In accordance with μPET and ex vivo autoradiography, a positive in vitro [^18^F]DPA-714 uptake was visible in the focal TBI lesions in mice with high initial NSS (Fig. 5a). As shown in Fig. 5b, staining for GFAP-positive astrocytes was enhanced in all TBI mice and the number of GFAP-positive cells correlated with NSS (Fig. 5d) and [^18^F]DPA-714 uptake (Fig. 5f).

The established neuropathological marker for axonal injury, SMI-32 [29, 31], identified strongly immunopositive axonal retraction bulbs in all TBI brains (Fig. 5c). Both TBI mice with focal brain lesions and mTBI mice with entirely diffuse damage showed damaged axons throughout the brain hemispheres. Again, axonal damage was worse in mice with more severe trauma and positive [^18^F]DPA-714 uptake in autoradiography (Fig. 5c, g). As expected [29], SMI-32 staining identified normal axonal filaments in the brains of sham-operated mice but no pathological staining of damaged axons (Fig. 5c). The degree of axonal damage correlated strongly with the initial severity of functional deficits (Fig. 5e).

## Discussion

In this study, we used μPET imaging with the specific TSPO ligand [^18^F]DPA-714 in a mouse closed head brain trauma model and correlated it with functional and histopathological trauma severity and reduced cerebral glucose uptake. By combining μPET imaging with ex vivo autoradiography and concomitant IBA-1 antibody staining on the same brain sections, we were able to identify activated microglia as the direct cellular correlate of the increased [^18^F]DPA-714 uptake.

We used a weight-drop brain injury model to generate a closed head injury in mice [20, 21, 23, 24]. The highly variable combination of both focal cortical contusions and diffuse axonal damage in this model reflects closely the heterogeneity of human TBI [33, 34]. Identical to the Glasgow Coma Scale in clinical TBI [35], outcome in our mouse model can be monitored on a relatively simple functional scale which correlated with postmortem histopathological analysis of trauma severity and in particular the presence of focal cortical lesions. Importantly, a positive uptake of the TSPO-tracer and a measurable deficit in glucose uptake in μPET were seen exclusively in mice with worse functional outcome and more severe trauma that included focal cortical damage.

The heterogeneous pattern of focal cortical contusions and global diffuse axonal damage in our TBI model made analysis of the imaging data challenging. Diffuse trauma lesions extended to different brain regions and so it was difficult to define an unaffected reference region. Furthermore, a truly genuine reference region with no TSPO expression does not exist because of the ubiquitous glial expression pattern of TSPO in the brain [8]. Cerebellum has been recently validated as a pseudo-reference region for TSPO binding radiotracers in Alzheimer patients [25] and in a mouse model of Alzheimer’s disease [26]. It has also been used as internal reference for assessment of brain metabolism with [^18^F]FDG PET in rat models of TBI [27, 28]. Assessment of cerebellar [^18^F]DPA-714 uptake in calculated mean SUVs for the outlined cerebellar ROIs in all mice was used in the present study to confirm this brain region as a relative reference region also in our model. Radiotracer binding in cerebellum showed no differences between the sham, mTBI, and TBI mice.

We used [^18^F]FDG-μPET to correlate TSPO ligand imaging to brain metabolism. Metabolic [^18^F]FDG imaging has not been previously reported in a mouse TBI model, but our findings match up with previous reports in rats even if in these studies, TBI model, trauma severity, and acquisition protocols and scan times [7] were different than those in the present study. In agreement with our results, an early but sustained reduction of glucose uptake in focally damaged brain areas is evident within the first days after trauma in rat models of TBI [27, 28, 36–38]. In our mouse study, reduced glucose metabolism was only visible in animals with focal cortical lesions and more severe trauma. In mice with solely diffuse axonal damage and less severe functional outcome score [^18^F]FDG accumulation was largely homogenous in the whole brain. However, metabolic imaging in the present study was not extended beyond day 5 and thus the late deterioration seen in some recent studies after mTBI in rats [37] was not captured in our study. Another limitation of the present study is that due to technical reasons, [^18^F]FDG-μPET imaging was not possible for all animals on one designated day but was performed on either on days 2, 4, or 5 after trauma. Therefore, a detailed analysis of the [^18^F]FDG-μPET scans on each day was not possible. Thus, the correlation analysis of [^18^F]FDG-μPET scans over 2–5 days may be inferred solely as a metabolic correlate for the consecutive [^18^F]DPA-714 imaging data on day 7.

Accumulation of [^18^F]DPA-714 by μPET was increased in the focal lesions on days 7 and 16 after TBI. In agreement with a previous study in rat TBI, no significant tracer uptake could be detected acutely 24 h after TBI [39]. Functional trauma severity strongly correlated with the increased [^18^F]DPA-714 accumulation in μPET on day 7. Again, no clear tracer accumulation could be detected at any time point in mice with mTBI, even if a slightly rising trend of [^18^F]DPA-714 uptake until day 16 was apparent. In a previous study [39] in rats, lesion to normal ratios of [^18^F]DPA-714 were increased as early as 2 days after controlled cortical impact injury. However, similar to our data, the ratios peaked around day 6 and then gradually decreased to nearly normal levels on day 28 [39]. In mouse models of ischemic stroke, temporal pattern of [^18^F]DPA-714 binding in μPET peaked around 10–14 days after stroke [11, 40] and a prominent binding of the radiotracer within the ischemic lesions persisted up to 16 days after the ischemic insult [40].

Ex vivo autoradiography subsequent to [^18^F]DPA-714-μPET imaging confirmed the in vivo μPET findings and showed a significant increase of TSPO ligand binding on days 7 and 16 after TBI. For quantitative analysis, the images of ex vivo autoradiography were aligned with IBA-1 stained brain sections. This permitted the identification of microglia as the [^18^F]DPA-714 binding cellular structures. The increased counts of IBA-1 positive microglia correlated strongly with the increased [^18^F]DPA-714 binding in ex vivo autoradiography. These data are in agreement with results of previous autoradiography studies in rats showing increased TSPO ligand binding and microglia activation by staining on adjacent brain sections 1 week after TBI [14, 39, 41, 42]. Similar findings have been reported after ischemic stroke in mice as [^18^F]DPA-714 binding strongly co-localized with IBA-1 positive microglia on adjacent brain sections 14 days after ischemic stroke [43]. An in vitro autoradiography study using [^3^H]PK11195 and a closed head weight-drop TBI model similar to ours [23, 44] also found a significant increase in TSPO ligand binding in the contusioned brain areas 7 days after TBI [44] though the radiotracer binding was not confirmed with a correlative immunohistochemical analysis. Thus, our study is the first to directly identify microglia as the cellular correlate of [^18^F] DPA-714 binding by combined in vivo μPET, ex vivo autoradiography, and IBA-1 immunohistochemistry in a mouse model of closed head injury.

Morphological activation and increased cell counts of IBA-1 positive cells were detected by immunohistochemistry also after mTBI indicating that this method is more sensitive than [^18^F]DPA-714 autoradiography to identify microglia activation at cellular level. The temporal pattern of microglia responses in our study matches those reported for trauma-induced expression of M1 phenotype of microglia in rats [30]. In this previous study, the cell counts of M1 microglia (IBA1+/CD16+) in the contusioned brain tissue peaked 7 days after a controlled cortical impact injury and the expression level remained elevated over 14 days. In contrast, the expression of M2 microglia (IBA-1+/CD206+) showed a peak at 5 days and then declined to control levels by day 14 [30]. Activated microglia, identified morphologically by swollen cell bodies and thicker, shrunken processes, were also shown to be the main cellular correlates of sustained TSPO expression in injured brain tissue after a fluid percussion injury in rats [42].

Even if activation of microglia plays an important role in the pathophysiology of TBI [30], damage and cellular responses are not restricted to one cellular substrate but include a plethora of complex pathologies including diffuse axonal injury and astrogliosis [33]. As expected, astrogliosis and diffuse axonal injury were clearly detectable after TBI also in our mice model and these cellular reactions correlated with functional outcome and trauma severity. Positive brain [^18^F]DPA-714 uptake in autoradiography also correlated with astrocyte activation and the extent of axonal injury even if the pattern of the [^18^F]DPA-714 signal in autoradiography did not entirely overlap the more diffuse areas of axonal damage and increased GFAP staining. Nevertheless, [^18^F]DPA-714 binding to activated astrocytes [39] cannot be excluded, even if upregulation of TSPO expression after TBI has been shown to be more prominent in microglia than in astroglial cells [14, 41, 42, 45].

## Conclusions

[^18^F] DPA-714 uptake in μPET/autoradiography correlated with trauma severity, brain metabolic deficits, and microglia activation after closed head TBI.

## Abbreviations

ANOVA, analysis of variance; CH_3_CN, acetonitrile; DAB, 3,3′-diaminobenzidine; [^18^F]FDG, 2-[^18^F]Fluoro-2-deoxyglucose; FORE, fourier rebinning; HPLC, high-performance liquid chromatography; IBA-1, ionizing calcium-binding adaptor molecule (polyclonal antibody); NHS, normal horse serum; OSEM 2D, 2D-ordered subsets expectation maximization; PBS, phosphate buffered saline; μPET, micro-positron emission tomography; p.i., post injection; ROI, region of interest; rp, reversed-phase; RT, room temperature; SD, standard deviation; SUV, standardized uptake value; TBI, traumatic brain injury; TSPO, translocator protein; [^18^F]DPA-714, N,N-diethyl-2-(2-(4-(2-[^18^F]fluoroethoxy)phenyl)-5,7-dimethyl-pyrazolo[1,5-α]pyrimidin-3-yl)acetamide; ^11^C, carbon-11; ^18^F, fluorine-18; ^18^O, oxygen-18
